# Patient-Controlled Intravenous Analgesia for Advanced Cancer Patients with Pain: A Retrospective Series Study

**DOI:** 10.1155/2018/7323581

**Published:** 2018-04-04

**Authors:** Zhiyou Peng, Yanfeng Zhang, Jianguo Guo, Xuejiao Guo, Zhiying Feng

**Affiliations:** Department of Pain Medicine, First Affiliated Hospital, School of Medicine, Zhejiang University, Hangzhou 310003, China

## Abstract

**Objective:**

To compare the efficacy and side effects of patient-controlled intravenous analgesia (PCIA) with hydromorphone, sufentanil, and oxycodone on the management of advanced cancer patients with pain.

**Methods:**

Patients allocated to receive PCIA between January 2015 and December 2016 were chosen for this study. After reviewing medical records, we verified if hydromorphone, sufentanil, or oxycodone for PCIA could equally provide effective pain relief. A numeric rating scale (NRS) of cancer pain was applied before PCIA, at 4 hours after PCIA, and at the discontinuation of PCIA. Secondary, the incidence of clinical side effects attributed to PCIA was observed.

**Results:**

A total of 85 medical records were reviewed. PCIA with hydromorphone (*n*=30), sufentanil (*n*=34), and oxycodone (*n*=21) was used for cancer pain management. PCIA successfully improved pain control in 97.6% of the patients. The most common side effects were constipation (11.8%), nausea (8.2%), and sedation (5.9%). Drug addiction, delirium, or respiratory depression associated with PCIA was not reported in this case series study. No significant intergroup difference was observed in NRS at any of the abovementioned time points. There was no significant difference of analgesic effect among the hydromorphone, sufentanil, or oxycodone.

**Conclusion:**

PCIA provided timely, safe, and satisfactory analgesia for advanced cancer patients with pain and may be useful for titration of opioids, management of severe breakthrough pain, and conversion to oral analgesia. There was no significant difference of analgesic effect and side effect among the hydromorphone, sufentanil, and oxycodone.

## 1. Introduction

With the increasing number of cancer patients, cancer pain has grown up to be a major public health problem all over the world. Despite recent advances in diagnosis and treatment, cancer pain continues to present a significant challenge to cope with. The World Health Organization (WHO) analgesic ladder is now the preferred protocol of choice all over the world. In clinical practice, this protocol works effectively and is prompt in controlling mild-to-moderate cancer pain initially.

As the disease progresses, alternate routes of administration are frequently used for advanced cancer patients with pain. Although primarily used in the treatment of acute postoperative pain, PCIA has been applied to advanced cancer patients with pain which allows the patient to individualize therapy by self-administrating predetermined doses of opioid analgesics. It was shown that PCIA can decrease the delayed opioid administration from the time requested, accompanied by rapidity and ease of dose titration and adaptability to the variable analgesic dosing needs [1]. Another advantage is that it helps to indicate whether the particular pain was opioid responsive or not. It was well known that many centers now offer PCIA for cancer patients to manage chronic pain [1, 2]. However, evidence of safety and efficacy of PCIA devices was limited to advanced cancer patients with pain.

The goal of this study was to retrospectively review the safety and the analgesic efficacy of hydromorphone, sufentanil, and oxycodone by PCIA in advanced cancer patients with pain.

## 2. Method

The study was approved by the ethics committee of the First Affiliated Hospital, School of Medicine, Zhejiang University. Patients who used PCIA for cancer pain management with sufentanil, hydromorphone, and oxycodone between January 2015 and December 2016 at the First Affiliated Hospital of the School of Medicine, Zhejiang University, were recruited in the retrospective cohort study. Criteria for exclusion include patient chart not available, discontinuation of PCIA therapy for more than 4 hours, and data not available. A total of 85 patients were identified and collected.

Information gathered from their electronic medical record included general demographic data, cancer diagnosis, date of PCIA pump used, 11-point (0 to 10) NRS before and after PCIA, times of breakthrough pain, the medications placed in the pump for basal and patient-controlled dosing, and opioid medications before and after pump placement.

Hospitalized cancer patients are usually treated by oncologists according to the National Comprehensive Cancer Network (NCCN) guidelines for opioid prescription. Whenever adequate pain control was not achieved with an amount of daily morphine equivalents ≥ 120 mg, they should get help from the department of pain medicine.

The decision criteria to install a PCIA were that the patient presented with severe breakthrough pain requiring at least five daily doses of systemic opioid rescue medication, unable to take oral medication, or pain control still not satisfied after oral drug titration in the last 24 h. The patient should not have a history of drug or alcohol abuse.

When using different forms of opioid drugs, the opioid equivalence dosage was compared using oral morphine equivalent dose [3–5]. The administration of opioid was launched with the basal infusion and a demand bolus dose. The bolus dose was fixed at 20% of the daily infusion dose approximately.

Discontinuation of therapy criteria is that pain was satisfactorily controlled for at least 24 hours or patients have clinical complications such as respiratory depression.

When the patient received PCIA as a supplementary rescue technique, their usual systemic medication was still using according to the NCCN guideline-based analgesic administration [6]. After the first 24 hours, opioid consumption in PCIA was added to the daily prescription.

Pain relief was defined as comfortable with subtotal pain relief with desired no increase of morphine dose. After pain relief, the patient took oral oxycodone or fentanyl transdermal patch equal to the intravenous requirement [7].

During the PCIA utilizing, a nurse assessed the baseline blood pressure, heart and respiratory rates, level of consciousness, pain score, and any other adverse effects.

Our primary outcome was to verify if different PCIA opioid-based solutions could effectively provide pain relief in cancer patients. In addition, the incidence of clinical side effects associated with PCIA therapy, including respiratory depression, drowsiness, constipation, and delirium was also observed.

Data were collected and analyzed using SPSS version 16.0 for Windows (SPSS Inc., Chicago, IL, USA). Continuous variables were presented as means ± standard deviation, and categorical data were shown as numbers and percentages. The independent sample *t*-test, the chi-square test, or the Mann–Whitney *U* test was utilized to group variables. *P* value less than 0.05 was considered significant.

## 3. Results

**Figure 1:**
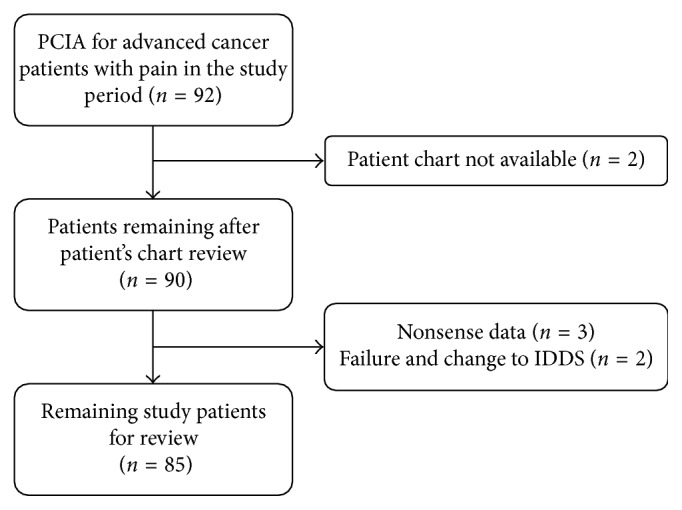
Study flowchart.

A total of 92 patients' records were included in this retrospective study. Screening of medical records was the first step to select eligible patients. Five were excluded due to missing data. PCIA failed in the remaining 2 patients, and intrathecal drug (morphine) delivery system was implanted for treatment of patients with intractable pain (Figure 1). PCIA with hydromorphone (*n*=30), sufentanil (*n*=34), and oxycodone (*n*=21) was used for cancer pain management. Depending upon the discontinuation criteria for PCIA established, success after PCIA prescription occurred in all of the patients except two patients. PCIA successfully improved pain control in 97.6% of the patients. Baseline characteristics of the patients are shown in Table 1.

A comparison of NRS score was showed before PCIA, at 4 hours after PCIA, and at the discontinuation of PCIA. Mean NRS score before PCIA in group hydromorphone, group sufentanil, and group oxycodone was 5.0 ± 1.1, 4.9 ± 1.0, and 5.3 ± 1.1. At 4 hours after PCIA, these decreased to 2.3 ± 0.8, 2.0 ± 1.0, and 2.2 ± 0.7. When PCIA is discontinued, these decreased to 1.9 ± 0.7, 1.7 ± 0.7, and 2.0 ± 0.6, respectively. No significant intergroup difference was observed in NRS at any of the abovementioned time points (Table 2).

PCIA therapy was employed for an average length of 5.9 ± 4 days. The most common side effects were constipation (11.8%), nausea (8.2%), and sedation (5.9%). Drug addiction, delirium, or respiratory depression originated in PCIA was not reported in this case series study (Table 3). There was no significant difference in side effect among hydromorphone, sufentanil, or oxycodone.

## 4. Discussion

The vast majority of advanced cancer patients with pain can obtain satisfactory control by following the WHO analgesic ladder with morphine sustained-release tablets, fentanyl transdermal system, and other noninvasive drug treatments [8]. However, some situations are still difficult to deal with, such as difficulty swallowing, recurrent nausea and vomiting with oral morphine sustained-release tablets, cannot tolerate fentanyl transdermal system, and frequent short bursts of breakthrough pain.

Application of PCIA can maintain the blood drug concentration near the lowest effective plasma concentration. The pharmacological characteristics of PCIA allowed for determining the treatment on needs of the patients. In addition, the effective blood concentration to maintain a stable and minimum effective quantity eliminates the difference to avoid the risk of drug overdose or insufficient. Compared to the conventional analgesic therapy, the PCIA is well accepted as a helpful and effective technique by the cancer patient with being able to control the pain themselves and the minimum delay between request for analgesia and pain relief [9–11].

The breakthrough pain was defined as a rapid onset, short duration, moderate-to-severe intensity, and frequent occurrence. The typical duration of an episode is 15–30 minutes. The frequency of pain episodes can vary from a single time to several times daily or weekly [12, 13]. With growing recognition of the prevalence and potential negative consequences of breakthrough pain, a short-acting drug is usually offered as needed during regular opioid treatment. Although the use of traditional oral opioid formulation and newer transmucosal fentanyl formulation are both valid options, a PCIA with pumps can also be used to allow treatment of breakthrough pain.

An obvious advantage is that PCIA can avoid delays in the administration of analgesics and benefits for patients suffering from frequent breakthrough pain episodes. PCIA has the advantages of immediately releasing the drug whenever the patient demands in low doses, short intervals, short duration of effect, and easy titration of opioids. Another one is that PCIA can ease of dose titration and adaptability to patients need to analgesics [14].

Hydromorphone, sufentanil, and oxycodone are opioid analgesics currently widely used in cancer pain. They are primarily an agonist at *μ* receptors which exert potent analgesic effects but also be coupled with adverse effects like nausea, vomiting, constipation, itching, and respiratory depression [3, 15]. The most common adverse reactions following PCIA were constipation (11.8%), nausea (8.2%), and sedation (5.9%), and no life-threatening adverse effects, such as respiratory depression, were recorded, even in older patients or when high doses were administered. Our result indicated that patients administering consistent doses of morphine with rapid modalities showed a minimal risk, if PCIA is done by skilled professionals in an adequate environment. In addition, opiate tolerance in patients who chronically receive relevant opioid doses for the management of cancer pain was considered for another reason.

Our experience suggests that hydromorphone, sufentanil, and oxycodone provides a good safety profile and represents an effective analgesic drug in cancer patient. All cancer patients treated with hydromorphone, sufentanil, and oxycodone of PCIA enjoyed satisfactory analgesia. The pain intensity score and times of breakthrough pain recorded during the treatment with PCIA indicated significant pain relief when compared to the starting time and remained stable for the entire time of treatment.

This study suffered from the limitations of any retrospective design. Some data records were not retrieved. Most detailed pain characteristics data such as movement related, light-dark intervals and sleep quality as was not in a position to be collected. In addition, the data of the effect of PCIA on anxiety, depression, and quality of life were insufficiency. Regardless of the fact that PCIA resulted in shorter length of hospital stay allowing to be available for other patients, the cost of drug and pump increases. The economic aspect of the use of PCIA is a worthwhile topic for further exploration.

In conclusion, PCIA, providing timely, safe, and satisfactory analgesia for advanced cancer patients with pain, may be useful for treating breakthrough pain and titration of opioid to aid weaning to oral analgesia. There was no significant difference of analgesic effect and side effect among the hydromorphone, sufentanil, and oxycodone. Clinicians should be familiar with the benefits and potential risks of PCIA with many kinds of opioids. More prospective, randomized controlled trials of clinical safety and efficacy of PCIA with opioid should be examined in advanced cancer patients with pain.

## Figures and Tables

**Table 1:** Patient characteristics.

	Hydromorphone	Sufentanil	Oxycodone
Number of patients	30	34	21
Male/female	18/12	18/16	13/8
Age (years)	59.8 ± 9.8	61.8 ± 9.4	59.9 ± 10.6
*Cancer location*			
Gastrointestinal	22	23	14
Bronchopulmonary	4	6	1
Urogenital	1	2	1
Others	3	3	5
Metastases present	28	33	19
*Main location of pain*			
Head/neck/upper extremity	2	2	1
Thorax	4	5	3
Abdomen-pelvic	20	24	14
Hip/lower extremity	3	1	2
Multiple sites of pain	1	2	1

**Table 2:** NRS scores of the two groups at different times in the three groups.

NRS	Hydromorphone	Sufentanil	Oxycodone
T1	5.0 ± 1.1	4.9 ± 1.0	5.3 ± 1.1
T2	2.3 ± 0.8	2.0 ± 1.0	2.2 ± 0.7
T3	1.9 ± 0.7	1.7 ± 0.7	2.0 ± 0.6

T1: before PCIA; T2: at 4 hours after PCIA; T3: at the discontinuation of PCIA.

**Table 3:** The incidences of adverse effect in the three groups.

	Hydromorphone (*n*=30)	Sufentanil (*n*=34)	Oxycodone (*n*=21)
Nausea/vomiting	3	2	2
Sedation	1	3	1
Constipation	5	5	3
Drug addiction	0	0	0
Respiration depression	0	0	0
Delirium	0	0	0
